# Median nerve swelling in RA patients: an 8-year longitudinal MRI-based study

**DOI:** 10.1186/s13244-026-02267-8

**Published:** 2026-04-22

**Authors:** Su Wu, Alex Wing Hung Ng, James Francis Griffith, Fan Xiao, Miaoru Zhang, Lai-shan Tam

**Affiliations:** 1 Department of Imaging and Interventional Radiology, The Chinese University of Hong Kong, Hong Kong, China; 2 Department of Medicine & Therapeutics, The Chinese University of Hong Kong, Hong Kong, China

**Keywords:** Rheumatoid arthritis, Carpal tunnel syndrome, Median nerve, MRI

## Abstract

**Objectives:**

Rheumatoid arthritis (RA) patients are prone to carpal tunnel syndrome (CTS). MRI can accurately detect median nerve swelling associated with CTS as well as evaluate synovial inflammation and structural damage. A median nerve cross-sectional area (CSA) of > 15 mm^2^ is the best MRI diagnostic criterion of CTS. This study investigates the prevalence of median nerve swelling in early RA patients, its relationship to inflammation and structural damage, and long-term outcome following treatment.

**Materials and methods:**

Retrospective study of early RA patients who underwent clinical, serology, radiography, and dynamic contrast-enhanced MRI of the wrist at baseline, year 1, and year 8. Median nerve cross-sectional area (CSA), median nerve enhancement and perfusion, retinacular bowing, synovial inflammation, structural damage and functional impairment were assessed.

**Results:**

81 early RA patients (age: 54 ± 13 years, F/M: 64/17) were studied. Undue median nerve swelling was present in 25 (31%) at baseline and 37 (46%) of 81 ERA patients at year 8. Undue median nerve swelling was moderately (r = 0.634) related to tenosynovitis volume at baseline but was otherwise not related to synovitis and structural damage at either baseline, year 1, or year 8. Median nerve swelling did not regress long-term. At year 8, CTS symptoms were present in about half of RA patients and were not related to median nerve swelling. Functional impairment at year 8 was more frequent in patients with median nerve swelling.

**Conclusion:**

Undue median nerve swelling is common in RA patients, is not related to synovitis or structural damage, does not regress with treatment, and is linked to long-term functional impairment.

**Critical relevance statement:**

Median nerve swelling, indicative of carpal tunnel syndrome, is common in RA patients, does not regress with reduction in synovitis or tenosynovitis after treatment and is associated with more severe and more frequent systemic functional impairment.

**Key Points:**

- Almost one-third of RA patients fulfilled MRI criteria for carpal tunnel syndrome (CTS) diagnosis at baseline, increasing to almost one-half of patients at year 8.
- Long-term median nerve swelling is not related to tenosynovitis, synovitis or structural damage.
- Functional impairment was over twice as common in patients with undue median nerve swelling than those without undue median nerve swelling.

**Graphical Abstract:**

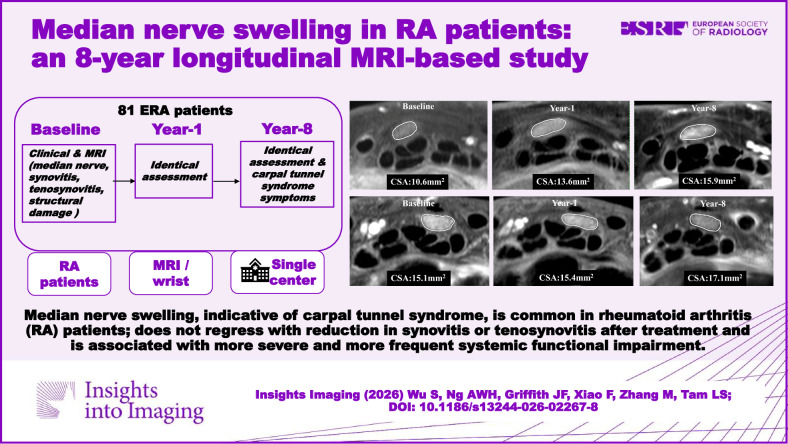

## Introduction

Rheumatoid arthritis (RA) is a well-known cause of carpal tunnel syndrome (CTS). CTS occurs in 1–4% of the normal population, but it is more common in RA patients [1–3]. Synovial proliferation and structural deformity are traditionally considered responsible for median nerve compression in RA patients, though no studies have been performed to test this assumption [4]. RA patients with CTS have more severe symptoms and worse hand function than those without CTS [1]. As there is considerable overlap in hand symptoms between CTS and RA, clinical assessment of CTS in RA patients is very limited, as CTS symptoms occur commonly in RA patients irrespective of whether CTS is present or not [2, 5–7]. Clinical tests for CTS, such as Tinel’s, Phalen’s, and Reverse Phalen’s test, are frequently positive in patients with wrist synovitis and/or tenosynovitis, severely limiting their specificity in identifying CTS in RA patients [2, 7]. As a result, greater reliance is placed on ancillary tests such as NCT, ultrasound (US), or MRI to diagnose CTS in RA patients [8–14].

Nerve conduction studies have a long examination time, are associated with patient discomfort, have a high false-negative rate and can only identify neural compression after neural function is deranged [15]. US and MRI have high accuracy for diagnosing CTS [16–19]. Both methods rely primarily on identifying undue median nerve swelling close to or within the carpal tunnel, with a diagnostic accuracy varying from 79% to 98% for CTS [9, 17, 20–22]. Median nerve swelling in CTS occurs at or proximal to the tunnel inlet and/or at or distal to the tunnel outlet rather than within the mid-portion of the carpal tunnel [12, 20, 22].On MRI, a median nerve swelling of > 15 mm^2^ is the best diagnostic criterion of CTS, with a sensitivity of 100%, specificity of 94%, and overall accuracy of 98% for diagnosing CTS [20]. More severe median nerve swelling is associated with more severe CTS symptoms [20, 23]. Increased retinacular bowing is the next most discriminatory US and MRI criterion of CTS [20]. Increased median nerve blood flow on color Doppler US is a specific but insensitive indicator of CTS [16, 24].

MRI can both semi-quantitatively and quantitatively evaluate RA-related inflammatory and structural damage parameters and their relationship to median nerve swelling. A group of RA patients was followed up for 8 years, with all patients undergoing identical MRI examination and clinical assessment at baseline, year 1, and year 8. The purpose of this study was to investigate (1) the prevalence of undue median nerve swelling (i.e., a median nerve CSA of > 15 mm^2^) in patients with RA at presentation and long-term, (2) the determinants of median nerve swelling, and (3) the relationship between undue median nerve swelling and CTS symptoms as well as functional impairment. The prevalence of median nerve swelling and its relationship to clinical features, synovial/tenosynovial proliferation, structural damage, and functional impairment were assessed.

## Materials and methods

Retrospective review of clinical and MRI data in ERA patients over an 8-year period. All patients in this cohort have been reported, focusing on long-term inflammatory and structural damage but not addressing median nerve changes [25]. The study protocol was approved by the local Ethics Committee, with signed informed consent obtained from all participants.

### Patients

131 patients were recruited at baseline. Patients were recruited if they fulfilled the 2010 American College of Rheumatology (ACR)/European League Against Rheumatism classification (EULAR) criteria for ERA, were treatment naïve at the study onset and were willing to participate in the study. Identical clinical and MRI assessments were performed at baseline, year 1, and year 8. 95 patients finished the year 1 assessment, and 81 patients finished the year 8 assessment. Patients who did not undergo a year 8 MRI examination were excluded from this study, as shown in the patient flowchart (Supplementary Fig. 1). Fifty patients were subsequently excluded as they were either unable to complete the year 8 assessment or had developed a serious concurrent illness, leaving 81 patients (64 females, 17 males, age 61 ± 13 years) in the final study cohort.

### Clinical assessment

**Fig. 1:**
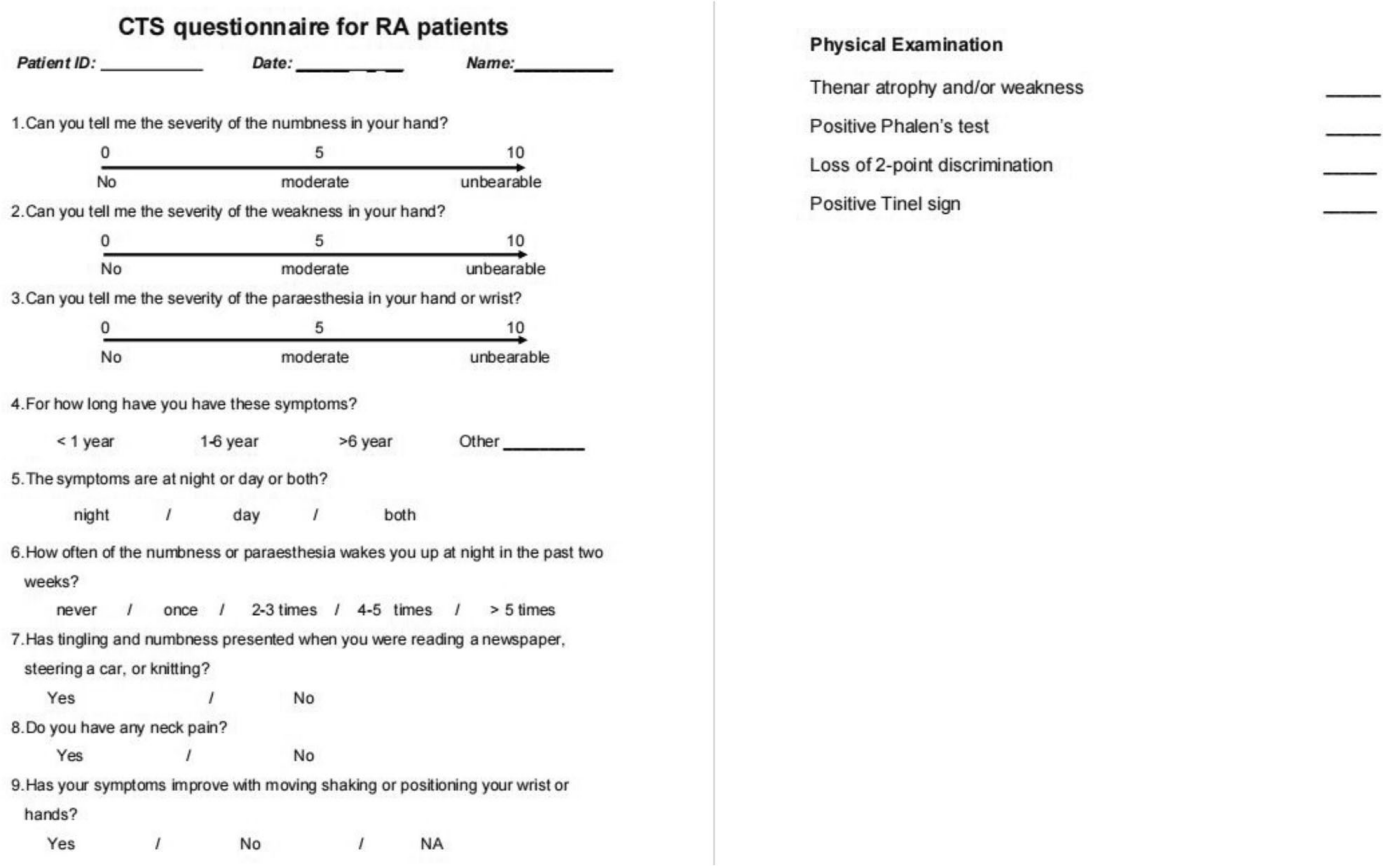
Self-designed CTA questionnaire for RA patients addressing 9 hand symptoms and 4 physical tests

Clinical assessments were performed at the Rheumatology Department. Clinical assessments included DAS28 (Disease Activity Score 28) and SDAI (Simple Disease Activity Index) to assess disease activity, as well as Health Assessment Questionnaire–Disability Index (HAQ-DI) to assess functional ability. Functional impairment was defined as a single HAQ of > 0.5 or an HAQ increase of > 0.35 compared to year 1 [26]. Serology (ESR, CRP) was also performed. At year 8 follow-up, four clinical tests (Phalen’s test; loss of 2-point discrimination, Tinel’s sign, and thenar muscle atrophy assessment) were performed on both wrists by a physician, and a specifically designed new CTS questionnaire was completed (Fig. 1).

### MRI assessment

At baseline, all patients underwent a standardized MRI examination of the most clinically affected wrist. At year 1 and year 8, the same wrist was examined by MRI. The scanning protocol is outlined in Supplementary Material 1.

### MRI analysis

All MRI measurements were made on a personal computer using dedicated DICOM image assessment software (OsiriX), following image zooming and contrast adjustment to optimize median nerve delineation. All measurements were undertaken by one of two musculoskeletal radiologists, one with 20 years and the other with 5 years of experience, both supervised by a musculoskeletal radiologist with 30 years of MR experience.

#### Median nerve caliber

**Fig. 2:**
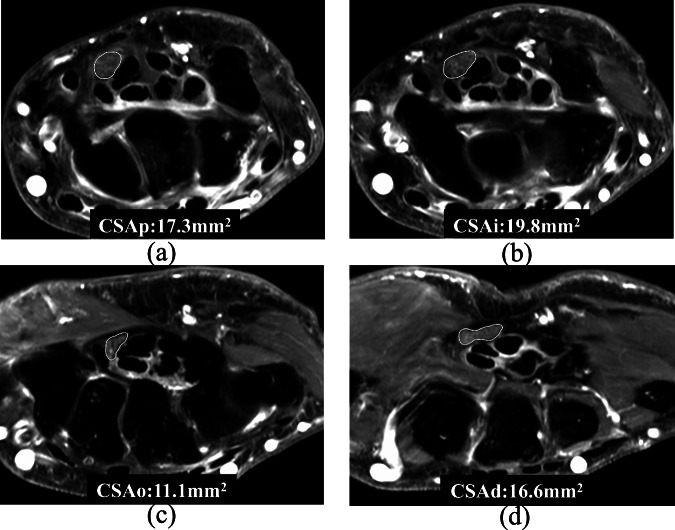
59-year-old female with mild tenosynovitis. Median nerve cross-sectional area (**a**) proximal to carpal tunnel inlet; (**b**) at carpal tunnel inlet; (**c**) at carpal tunnel outlet; and (**d**) distal to carpal tunnel outlet

**Fig. 3:**
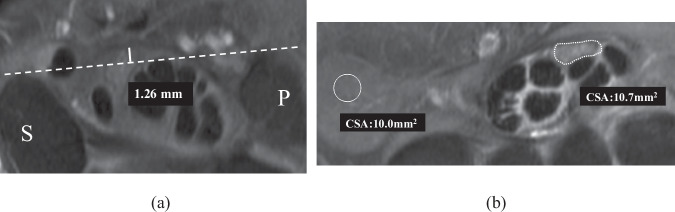
**a** A 47-year-old female with moderate hand numbness and weakness. Tangential line (dashed line) connecting volar surfaces of the pisiform (P) and scaphoid (S) bones, Perpendicular line (solid line) between the tangential line and the undersurface of the transverse carpal ligament. In this case, retinacular bowing at the tunnel inlet (Bri) = 1.26 mm. **b** A 10 mm^2^ ROI is placed within the hypothenar muscle to measure the muscle signal intensity and median nerve outlined to measure median nerve signal intensity. Signal intensity ratio (SI) is the mean signal intensity of the median nerve divided by that of the hypothenar muscle. In this case, SI ratio = 1.07

Median nerve caliber measurements were made on post-contrast T1 fat-suppressed sequences. Median nerve cross-sectional area (CSA) was measured at four locations, namely proximal to the tunnel inlet (CSAp); at the tunnel inlet (CSAi); at the tunnel outlet (CSAo); and distal to the tunnel outlet (CSAd) (Fig. 2). ‘Carpal ‘tunnel inlet’ and ‘tunnel outlet’ were defined as immediately deep to the proximal and distal margins of the flexor retinaculum respectively. ‘Proximal to the tunnel’ was immediately proximal to the flexor retinaculum, while ‘distal to the tunnel’ was immediately distal to the flexor retinaculum. Retinacular bowing, measured at the tunnel inlet (Bri) and outlet (Bro) levels, were defined as the perpendicular distance between the deep margin of the retinaculum and a tangential line drawn between the volar aspects of the scaphoid and pisiform bones at the tunnel inlet and the trapezium and hook of hamate at the tunnel outlet (Fig. 3a). A median nerve CSA of > 15 mm^2^ at any location was considered as ‘undue median nerve swelling’ [19].

#### Median nerve contrast enhancement

Relative median nerve enhancement was measured on post-contrast T1WI fat-suppressed images. A circular region of interest (ROI) was placed in the middle of the hypothenar muscle (10 mm^2^ in size), and the median nerve and signal intensity ratio (SI) between the median nerve and muscle were calculated (Fig. 3b).

#### Median nerve perfusion

On dynamic contrast-enhanced MR images, an ROI was placed in the middle of the median nerve, and perfusion curves were drawn, which were categorized as one of three different types, namely: A: fast enhancement followed by gradual increase, B: slow enhancement followed by gradual increase, and C: unclassified type [27].

#### Semi-quantitative and quantitative assessment of inflammatory and structural damage

Rheumatoid arthritis magnetic resonance imaging score (RAMRIS) was used to semi-quantitatively measure inflammatory and structural damage (erosions and joint space narrowing) parameters at baseline, year 1, and year 8. Tenosynovial and synovial proliferation was also measured quantitatively by manually demarcating the tenosynovitis and synovitis areas on each consecutive image and summating synovitis and tenosynovitis areas to obtain tenosynovial and synovial volume (Supplementary Fig. 2).

#### Reliability test

Two musculoskeletal radiologists (with 21 years and 3 years of MR reporting experience) independently measured medial nerve CSA (at the four designated locations) and bowing retinaculum (at the two designed locations) in 30 patients on both PDFS and T1FS + C sequences. Results were compared to calculate the interclass correlation coefficient. Two weeks later, one reader repeated all the initial measurements.

### Statistical analysis

SPSS software (SPSS for Microsoft Windows, version 24.0, IBM) was used for data analysis. For normally distributed data, the mean and standard deviation were calculated. For other data, median and interquartile range (IQR) were used. Repeated ANOVA was used to ascertain differences in MRI measurements at baseline, year 1, and year 8. Chi-square test was used to compare the percentage of RA patients who fulfilled MRI criteria for CTS at baseline, year 1, and year 8. Spearman’s correlation test was used to find the relationship between median nerve CSA, synovial inflammation, structural damage, and clinical symptoms. Interclass correlation was used to assess inter- and intra-observer agreement for MRI parameter measurements. A value of 0.20 or less implied poor agreement; 0.21–0.40 fair agreement; 0.41–0.60 moderate agreement; 0.61–0.80 substantial agreement; and 0.81–1.00 excellent agreement. Median nerve parameters measured on different sequences were compared, and the coefficient of variation was calculated.

## Results

### Reliability testing

Excellent inter- and intra-observer (range: 0.84–0.97) agreement was found for all MRI parameters measured. Median nerve CSA measurements on PDFS and T1FS + C sequences showed excellent (range: 0.90–0.99) agreement.

### Prevalence of carpal tunnel symptoms and signs

#### Clinical symptoms

Fifty-two (64%) of 81 patients at year 8 had at least one CTS-related symptom. Numbness, hand weakness, and hand paresthesia were present in 43 (53%), 36 (44%), and 35 (43%) of 81 patients, respectively, at year 8 (Table 1).

**Table 1:** Clinical, MRI assessment and clinical examination findings in patients who did not fulfill and who fulfilled the MR CTS criterion, defined as a median nerve cross-sectional area (CSA) of > 15 mm^2^ at year 8

	Not-fulfilled (*n* = 44)	Fulfilled (*n* = 37)	*p*-value
Age (years)	59 ± 14	64 ± 9	**0.007**
Sex (F/M)	40/4	27/10	**0.043**
Disease duration (years)	8.3 ± 1.7	7.9 ± 2.0	0.169
DM	3 (6.8%)	2 (5.4%)	0.670
Clinical assessment			
Disease activity score 28	2.4 ± 1.0	2.9 ± 1.3	0.062
Simple disease activity index	7.2 ± 7.2	10.7 ± 8.8	0.140
CRP	4.2 ± 6.8	11.2 ± 19.0	**0.023**
ESR	32.9 ± 20.6	44.4 ± 35.7	0.098
MRI assessment			
Synovitis volume (cm^3^)	3.2 ± 3.2	3.2 ± 2.8	0.588
Tenosynovitis volume (cm^3^)	0.3 ± 0.6	0.4 ± 0.7	0.513
Total volume (cm^3^)	3.5 ± 3.4	3.6 ± 3.2	0.642
Bone erosion score	16.0 ± 19.3	12.5 ± 15.0	0.380
JSN score	9.0 ± 12.3	5.7 ± 8.5	0.169
Functional parameter			
HAQ score	0.3 ± 0.4	0.5 ± 0.5	0.102
Functional impairment	8 (18%)	18 (49%)	**0.003**
Symptom presence (*n*/%)			
Numbness	28 (61.4%)	15 (40.5%)	**0.038**
Weakness	22 (50.0%)	14 (37.8%)	0.273
Paresthesia	17 (38.6%)	18 (48.6%)	0.365
Clinical examination			
Thenar muscle atrophy, weakness	13 (29.5%)	15 (40.5%)	0.300
Phalen’s test	11 (25.0%)	11 (29.7%)	0.634
Loss of 2-point discrimination	16 (36.4%)	13 (35.1%)	0.909
Tinel’s sign	1 (2.3%)	2 (5.4%)	0.457

Older patients and male patients tended to have median nerve swelling. Functional impairment was over twice as prevalent in patients who fulfilled the MR criteria for CTS. Most patients tended to have symptoms and signs of CTS, and this was not necessarily related to a median nerve CSA of > 15 mm^2^ on MR

*DM* diabetes mellitus, *CRP* C-reactive protein, *ESR* erythrocyte sedimentation rate, *JSN* joint space narrowing, *HAQ* health assessment questionnaire

Bold numbers represent statistically significant probailities

#### Clinical signs

Loss of 2-point discrimination, thenar muscle atrophy ± weakness, and a positive Phalen’s sign were present in 29 (36%), 28 (35%), and 22 (27%) of 81 patients at year 8 (Table 1).

### Prevalence of median nerve swelling, enhancement, perfusion and retinacular bowing

#### Undue median nerve swelling

**Fig. 4:**
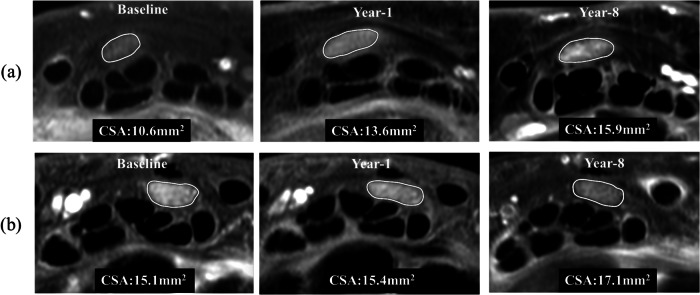
**a** A 63-year-old female median nerve cross-sectional area proximal to carpal tunnel inlet (CSAp) at baseline, year 1, and year 8. In this patient, CSAp increased from 10.6 to 15.9 mm^2^ at year 8. **b** A 53-year-old patient with CSAp median nerve swelling at baseline (15.1 mm^2^), which remained swollen (17.1 mm^2^) at year 8

25 (31%) of 81 ERA patients had median nerve swelling of > 15 mm^2^ (16.6 ± 3.7) at baseline. The median nerve remained swollen in all these 25 patients at year 8 (18.4 ± 6.2) (Fig. 4a). Twelve additional patients, i.e., a total of 37 (46%) of 81 ERA patients, had median nerve swelling of > 15 mm^2^ at year 8 (Fig. 4b) (Table 2). On average, median nerve CSA increased by 5–7% between baseline and year 8 on MR examinations, both proximal to (*p* = 0.035) and at the tunnel inlet level (*p* = 0.003) (Table 2).

**Table 2:** Patient characteristics and median nerve measurements at baseline, year 1 and year 8

	Baseline	Year 1	Year 8	*p*-value
	*n* = 81	*n* = 64	*n* = 81	
Age (years)	54 ± 13	56 ± 12	61 ± 13	**< 0.001**
Sex (F/M)	67/14	52/12	67/14	0.523
MN caliber (mm^2^)				
CSAp	13.4 ± 3.6	13.2 ± 4.0	14.4 ± 4.9	**0.035**
CSAi	13.3 ± 3.2	13.0 ± 3.4	14.0 ± 4.0	**0.003**
CSAo	12.1 ± 3.0	12.3 ± 2.5	12.6 ± 3.2	0.145
CSAd	12.2 ± 3.9	12.4 ± 3.3	13.0 ± 4.4	0.433
Bri	2.9 ± 1.4	2.3 ± 1.1	2.2 ± 1.5	**0.004**
Bro	0.7 ± 0.8	0.2 ± 0.9	0.5 ± 1.0	**0.011**
SI ratio	1.0 ± 0.2	1.0 ± 0.1	1.0 ± 0.2	0.070
MN perfusion (*n*/%)				
Type a	39 (48.1%)	25 (39.1%)	43 (53.1%)	0.150
Type b	12 (14.8%)	14 (21.9%)	18 (22.2%)	0.450
Type c	26 (32.1%)	25 (39.1%)	16 (19.8%)	0.150
MN swollen*	25 (30.9%)	21 (32.8%)	37 (45.8%)	0.117
MRI quantitative assessment				
Synovitis volume (cm^3^)	5.5 ± 4.4	2.4 ± 2.7	3.2 ± 3.0	**< 0.001**
Tenosynovitis volume (cm^3^)	1.2 ± 1.5	0.2 ± 0.5	0.4 ± 0.7	**< 0.001**
Total volume (cm^3^)	6.7 ± 5.0	2.6 ± 2.9	3.6 ± 3.3	**< 0.001**
MRI semi-quantitative assessment				
Synovitis score	5.9 ± 3.1	3.5 ± 2.5	4.1 ± 2.7	**< 0.001**
Tenosynovitis score	3.9 ± 3.6	1.4 ± 2.0	1.7 ± 2.3	**< 0.001**
Bone erosion score	9.4 ± 9.6	10.1 ± 12.3	14.3 ± 17.4	**< 0.001**
JSN score	3.3 ± 7.0	3.3 ± 7.2	7.4 ± 10.8	**< 0.001**

The median nerve caliber increased slightly in size proximal to the tunnel (CSAp) and at the tunnel inlet (CSAi) between baseline and year 8. Bowing of the retinaculum at both the inlet and outlet reduced slightly over this period. A higher percentage of patients at year 8 met the MRI criteria for CTS (with a medial nerve caliber of > 15 mm^2^) than at baseline

*MN* median nerve, *CSA* cross-sectional area, *Br* bowing retinaculum, *SI* signal intensity

* Defined as > 15 mm^2^

Bold numbers represent statistically significant probailities

#### Median nerve enhancement

Relative median nerve enhancement at baseline was slightly higher (ratio 1.1 ± 0.2) in patients with undue median nerve swelling compared to patients without undue median nerve swelling (ratio 1.0 ± 0.2, *p* = 0.044). Similar changes were apparent in year 1 and year 8 (Table 2). Relative median nerve enhancement did not change between baseline and year 8 (Table 2).

#### Median nerve perfusion

Median nerve perfusion was similar for patients with and without undue median nerve swelling at baseline. Type A enhancement was more common in patients with undue median nerve swelling at year 1 and year 8. At year 1, 11 (52%) of 21 patients with undue median nerve swelling had type A enhancement (*p* = 0.020). At year 8, 25 of 37 (68%) patients with undue median nerve swelling had type A enhancement (*p* = 0.015).

#### Retinacular bowing

Retinacular bowing at both the tunnel inlet and outlet decreased by about 25% from baseline to year 8 (Table 2).

### Inflammatory and structural damage parameters

Semi-quantitative and quantitative assessment methods both showed that inflammatory parameters significantly decreased from baseline to year 1 and slightly increased from year 1 to year 8. Average tenosynovitis and synovitis RAMRIS scores were 3.9 and 5.9 at baseline, decreasing to 1.4 and 3.6 at year 1 and increasing slightly at year 8 (Table 2).

Tenosynovitis volume decreased from 1.2 cm^3^ at baseline to 0.2 cm^3^ at year 1 and increased to 0.4 cm^3^ at year 8, while synovitis volume decreased from 5.5 cm^3^ at baseline to 2.4 cm^3^ at year 1 and increased to 3.2 cm^3^ at year 8 (Table 2).

Structural damage parameters (bone erosion score and joint space narrowing score) changed little between baseline and year 1 but significantly increased between year 1 and year 8 (Table 2).

### MRI determinants of median nerve swelling

At baseline, tenosynovial volume was moderately (r = 0.634, *p* < 0.05) associated with median nerve CSA proximal to the tunnel level and weakly associated at other levels (r = 0.210–0.275, *p* < 0.05). No correlation between tenosynovitis severity and undue median nerve swelling was found at year 1 and year 8 (Supplementary Table 1). The severity of synovitis, whether assessed semi-quantitatively or quantitatively, was not related to undue median nerve swelling at baseline, year 1, or year 8 (Supplementary Table 1). Similarly, the severity of bone erosion or joint space narrowing was not related to undue median nerve swelling at baseline, year 1, and year 8 (Table 1). No correlation between tenosynovitis severity and undue median nerve swelling was found at year 1 and year 8 (Table 1). No correlation was found between diabetes and undue medial nerve swelling (*p* = 0.670, Table 1).

### Association between median nerve swelling and clinical symptoms, functional impairment

Hand numbness was less prevalent (41%) in patients with undue median nerve swelling compared to patients without undue median nerve swelling (61%) (Table 1). All other symptoms were similar in patients with and without undue median nerve swelling (Table 1).

Mean HAQ score was 0.5 in patients with undue median nerve swelling at year 8 compared to 0.3 in patients without median nerve swelling (*p* = 0.102). Functional impairment was present in 18 of 37 (49%) patients with undue median nerve swelling at year 8 compared to 8 of 44 (18%) without median nerve swelling (*p* = 0.003) (Table 1).

## Discussion

The first notable finding of this study is the high prevalence of CTS-related symptoms in RA patients. About half of RA patients had symptoms and signs of CTS at year 8. No correlation was found between undue median nerve swelling and CTS symptoms, emphasizing the limitations of clinical symptoms and signs in diagnosing CTS in RA patients [5, 8]. The second main finding of this study is the high prevalence of undue median nerve swelling at baseline, which became even more prevalent at year 8. Almost one-third (31%) of ERA patients had undue (> 15 mm^2^) median nerve swelling at baseline, which increased to almost one-half (46%) at year 8. This prevalence of undue median nerve swelling, consistent with CTS, is comparable to ultrasound-based studies of RA patients in which undue median nerve swelling prevalence varied from 15% to 51% (Table 3). The third main finding is that median nerve swelling was related to flexor tenosynovitis only at presentation. Moderate correlation was found between tenosynovial volume and median nerve swelling proximal to the tunnel inlet at baseline. Median nerve swelling was not related to tenosynovitis volume at year 1 or year 8 and was not related to synovitis or structural damage severity either at baseline, year 1 and year 8.

**Table 3:** Published studies focusing on carpal tunnel syndrome prevalence in RA patients

Reference	CTS prevalence	Diagnosis based on	Criteria (US) used to diagnose CTS
Kerasnoudis [8]	59 (51%) of 116 patients	US + NCS	MN CSA > 13 mm^2^ in carpal tunnel area Forearm: tunnel MN CSA ratio > 1.5
Dede [35]	43 (35%) of 122 patients	Clinical symptoms + US	Carpal tunnel inlet-forearm MN CSA difference Carpal tunnel inlet MN CSA (> 10.5 mm^2^) Carpal tunnel inlet MN medial-lateral diameter Carpal tunnel inlet to-forearm MN CSA ratio
Subasi [17]	21 (30%) of 70 patients	US + NCS	Tunnel inlet MN CSA > 10.5 mm^2^
Yagci [29]	23 (38%) of 60 patients	US	Tunnel inlet MN CSA > 10 mm^2^
Karadag [36]	30 (15%) of 200 patients	US ± NCS	CTS symptoms and MN CSA proximal to the tunnel inlet > 13 mm^2^ Positive NCS test and MN CSA proximal to tunnel inlet > 10 mm^2^

*US* ultrasound, *NCS* nerve conduction study, *MN* median nerve, *CSA* cross-sectional area

The fourth main finding relates to the relationship between median nerve swelling and patient function. About one in every two RA patients had undue median nerve swelling at year 8, and functional impairment, as evidenced by a reduced HAQ score, was present in half of these patients compared to only one-fifth of patients without undue median nerve swelling. In other words, at year 8, about one-third of RA patients overall had systemic functional impairment, and in most of these patients, this systemic functional impairment was associated with undue median nerve swelling.

All prior studies addressing median nerve swelling in RA patients used ultrasound assessment at a single time point and did not address longitudinal change in median nerve swelling or its relationship to tenosynovitis, synovitis, or structural damage (Table 3). The current study is the first to use MR assessment and the first to longitudinally follow changes in median nerve swelling in RA patients, enabling the relationship between median nerve swelling and tenosynovitis/synovitis, as well as structural damage severity, to be accurately assessed. From baseline to year 8, although tenosynovitis and synovitis significantly improved, median nerve swelling became more prevalent. Once median nerve swelling is present, it does not regress. This finding is clinically relevant as it has hitherto been considered that median nerve compression will improve as tenosynovitis and synovitis improve with treatment [5, 16, 28]. As such, other unrecognized causes, rather than tenosynovitis, synovitis, or structural damage, are most likely to account for increased long-term median nerve swelling in RA patients [29, 30]. Based on the results of this study, two possibilities are likely, namely (1) inherent median nerve changes, due to chronic compressive neuropathy, as reflected by more pronounced median nerve enhancement and faster median nerve perfusion in patients with undue median nerve swelling, at year 1 and year 8, compared to those without undue median nerve swelling [31, 32], and/or (2) reduced elasticity or increased fibrosis of the flexor retinaculum as evidenced by slight decrease in retinacular bowing from baseline to year 8. Also, regarding the increase in the degree of functional impairment seen in patients with undue median nerve swelling, it should be noted that the functional assessment HAQ addresses systemic rather than hand/wrist functional impairment. As such, systemic factors associated with median nerve swelling are more likely to lead to functional impairment in RA patients rather than local changes in the hands and wrists [33]. Median nerve caliber is not routinely assessed on wrist MRI examinations in RA patients [34]. Based on the results of this study, we recommend that it should be routinely assessed and measured at that location where the median nerve appears visibly largest, as this provides an indicator of co-existent CTS, not likely to improve on current standard treatment, and is associated with systemic long-term functional impairment. New studies should investigate further the causes of median nerve swelling in RA patients and its association with long-term systemic functional impairment.

This study has some limitations. First, about one-third of the original cohort did not return for the year 8 assessment, as some had died, had developed malignancy, or had moved away from the area. As no difference in baseline parameters was found between patients who completed and did not complete the year 8 review, this reduction in cohort size did not knowingly influence our results. Second, while median nerve measurements were available at baseline, year 1, and year 8, CTS symptoms and signs were only investigated at year 8. Third, nerve conduction tests were not performed, and other confounding factors such as body mass index, occupation, and thyroid function were not assessed.

In conclusion, CTS-related symptoms are common in RA patients and are not usually associated with undue median nerve swelling. Undue median nerve swelling, indicative of CTS, is present in about one-third of ERA patients at presentation and about one-half of patients at year 8. Undue median nerve swelling is moderately related to flexor tenosynovitis at presentation, though otherwise it is not related to tenosynovitis, synovitis, or structural damage. Median nerve swelling does not regress with treatment and is associated with more frequent functional impairment in the long term.

## Supplementary information

ELECTRONIC SUPPLEMENTARY MATERIAL

## Data Availability

The data underlying this article will be shared on reasonable request to the corresponding author.

## Abbreviations

CSA: Cross-sectional area
CTS: Carpal tunnel syndrome
ERA: Early rheumatoid arthritis
RAMRIS: Rheumatoid magnetic resonance imaging score
ROI: Region of interest
