# Supplementary material for: Median nerve swelling in RA patients: an 8-year longitudinal MRI-based study
Source: Insights Imaging. 2026 Apr 22;17:113. doi: 10.1186/s13244-026-02267-8 (PMC13103215; doi:10.1186/s13244-026-02267-8)
Supplement: Supplementary file 1 — ELECTRONIC SUPPLEMENTARY MATERIAL [file 13244_2026_2267_MOESM1_ESM.pdf]

Median nerve swelling in RA patients: an 8-year longitudinal MRI-based study

ELECTRONIC SUPPLEMENTARY MATERIAL

Supplementary Figure 1:

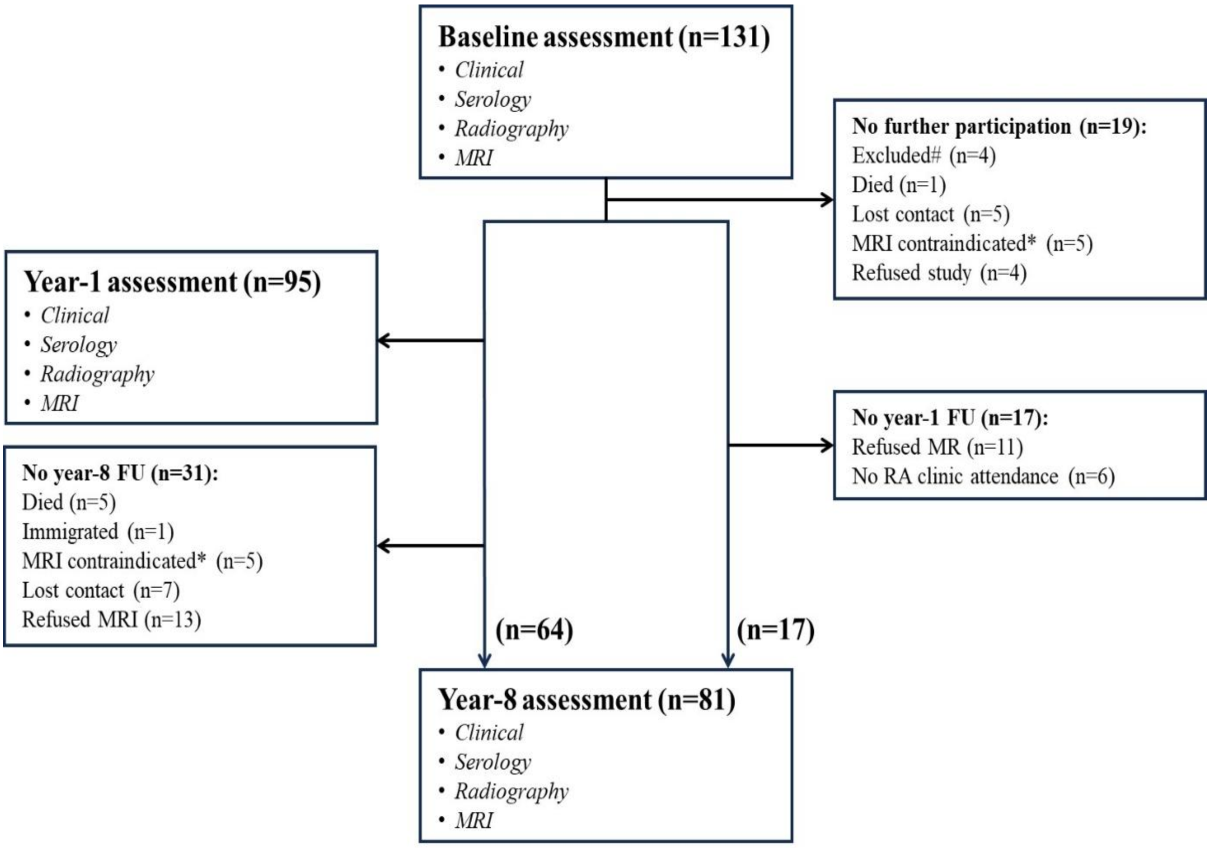

Flowchart of patient enrolment at baseline, year-1 and year-8. Sixty-four patients completed the baseline, year-1 and year-8 assessments. 17 patients did not undergo year-1 follow-up. #Excluded at year-1 due to diagnosis of systemic lupus erythematosus (n=3) and peripheral spondylarthritis (n=1) * Contrast enhanced MRI was contradicted because of implant (n=3), claustrophobia (n=1), malignant tumour (n=4), kidney failure (n=1), and age more than 90 years (n=1). There was no difference in clinical, serological, radiographic or MRI parameters at baseline between the 50 patients who did not complete and the 81 patients who did complete the year-8 follow up (*p* > 0.05 for all).

Supplementary Material 1:

MRI protocol:

Wrists were scanned in a Superman position on a 3.0-T system (Phillips Integra, Best, Netherlands) using a dedicated wrist coil and a scan plane extending from the distal radio-ulnar joints to the metacarpal bases. The following sequences were obtained: fat-saturated T1-weighted axial (TR 692ms; TE 12ms; echo spacing 12ms; flip angle 90°; FOV 80×80mm; slice thickness 3mm; NEX 2); fat-saturated T2-weighted coronal (TR 3121ms; TE 70ms; echo spacing 10ms; flip angle 90°; FOV 80×80mm; slice thickness 1.5mm; NEX 1); T1-weighted coronal (TR 547ms; TE 12ms; echo spacing 12ms; flip angle 90°; FOV 80×80mm; slice thickness 1.5mm; NEX 1); fat-saturated post-contrast T1-weighted axial (TR 692ms; TE 12ms; echo spacing 12ms; flip angle 90°; FOV 80×80mm; slice thickness 3mm; NEX 2). Dynamic contrast enhanced MRI (DCE MRI) was also performed (TR 3.8ms; TE 2.3ms; echo spacing 59.7ms; flip angle 12°; FOV 100 × 82mm; slice thickness 5mm; NEX 2) utilizing a pump injector and intravenous gadolinium contrast dose of 0.1mmol/kg.

Supplementary Figure 2:

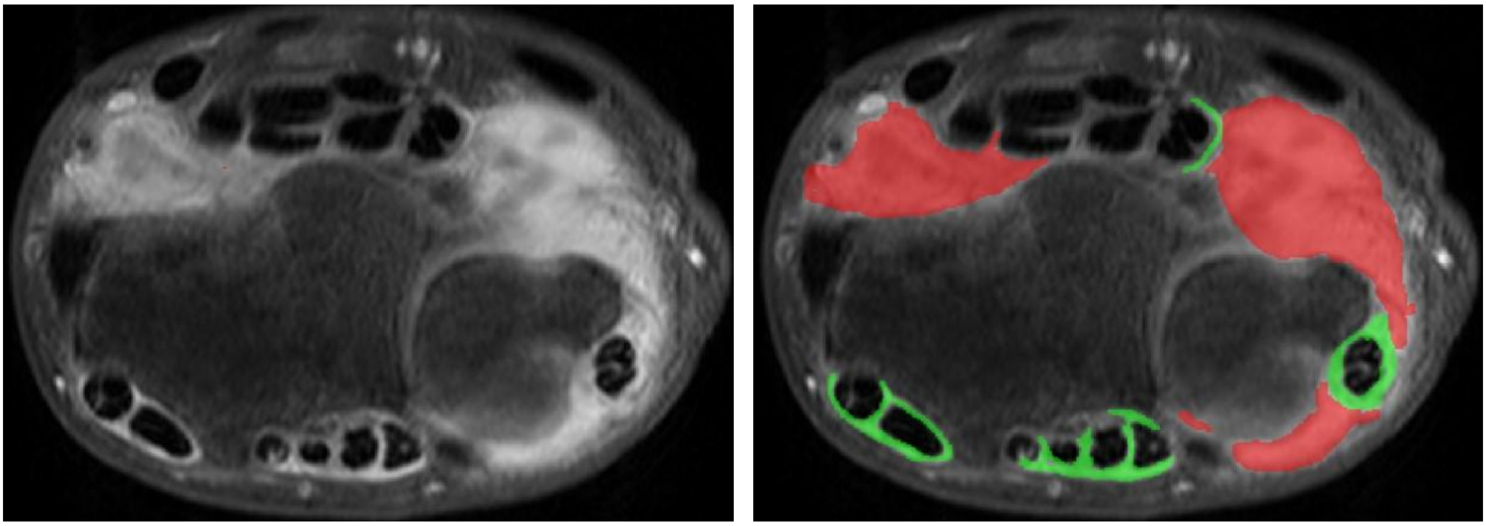

(a) (b)

Supplementary Figure 2: (a) 61-year-old female with severe wrist synovitis and mild tenosynovitis on post-contrast axial T1-weighted MR images with fat-suppression. (b) Regions of synovitis (red) and tenosynovitis (green) were manually segmented on serial images and summated. Synovitis volume: 14.6 cm^3; tenosynovitis volume: 2.7 cm^3

Supplementary Table 1: Correlation between synovial volume. tenosynovial volume, and median nerve CSA

	Synovial volume	Tenosynovial volume	Total volume
Baseline			
CSAp	r=0.176 p=0.053	**r=0.634 *p*=0.001**	**r=0.213 p=0.019**
CSAi	r=0.110 p=0.228	**r=0.275 *p*=0.002**	r=0.164 p=0.072
CSAo	r=0.060 p= 0.511	**r=0.208 *p*=0.022**	r=0.106 p=0.245
CSAd	r=0.037 p=0.687	**r=0.210 *p*=0.021**	r=0.011 p=0.903
Bri	r=0.099 p=0.280	r=0.131 p=0.154	r=0.121 p=0.185
Bro	**r=0.270 p=0.003**	**r=0.264 p=0.004**	**r=0.290 p=0.001**
Year-1			
CSAp	r= 0.055 p=0.611	r=0.022 p=0.839	r=0.061 p=0.572
CSAi	r=0.004 p=0.971	r=0.113 p=0.293	r=0.030 p=0.779
CSAo	r=0.055 p=0.612	r=0.103 p=0.339	r=0.059 p=0.580
CSAd	r=0.040 p=0.708	r=0.041 p=0.703	r=0.051 p=0.632
Bri	r=0.095 p=0.376	r=0.021 p=0.844	r=0.078 p=0.467
Bro	r=0.083 p=0.441	r=0.189 p=0.080	r=0.078 p=0.469
Year-8			
CSAp	r=-0.068 p=0.566	r=0.018 p=0.124	r=-0.021 p=0.859
CSAi	r=-0.121 p=0.304	r=0.091 p=0.439	r=-0.097 p=0.411
CSAo	r=0.109 p=0.355	r=0.155 p=0.188	r=0.147 p=0.212
CSAd	r=0.088 p=0.458	r=0.147 p=0.212	r=0.117 p=0.320
Bri	r=-0.068 p=0.567	r=0.005 p=0.964	r=-0.056 p=0.634
Bro	r=-0.043 p=0.715	r=0.125 p=0.290	r=-0.023 p=0.849
Symptoms at year-8			
numbness	r=0.082 p=0.488	**r=0.237 p=0.042**	r=0.117 p=0.321
weakness	r=0.165 p=0.159	**r=0.229 p=0.050**	r=0.175 p=0.135
paraesthesia	r=0.197 p=0.093	r=0.181 p=0.124	r=0.181 p=0.123
Total score	r=0.177 p=0.131	**r=0.233 p=0.046**	r=0.188 p=0.109
